# The evolving role of positron emission tomography in precision prostate cancer biopsy

**DOI:** 10.1111/eci.70062

**Published:** 2025-04-23

**Authors:** Alessio Imperiale, Valentina Berti, Roberto Luigi Cazzato, Darejan Mamulashvili Bessac, Alice Ressault, Luca Vaggelli, Mehdi Helali, Camilo Garcia, Hervé Lang, Desirée Deandreis, Luc Mertz, Afshin Gangi

**Affiliations:** ^1^ Nuclear Medicine University Hospitals of Strasbourg, University of Strasbourg Strasbourg France; ^2^ Nuclear Medicine and Molecular Imaging ICANS Strasbourg France; ^3^ IPHC, UMR 7178, CNRS/Unistra Strasbourg France; ^4^ Nuclear Medicine Careggi University Hospital Florence Italy; ^5^ Experimental and Clinical Biomedical Sciences ‘Mario Serio’ Florence University Florence Italy; ^6^ Interventional Radiology Strasbourg University Hospitals, Strasbourg University Strasbourg France; ^7^ Radiopharmacy Strasbourg University Hospitals, Strasbourg University Strasbourg France; ^8^ Nuclear Medicine Institut Gustave‐Roussy, Université Paris‐Saclay Villejuif France; ^9^ Urology Strasbourg University Hospitals, Strasbourg University Strasbourg France; ^10^ Radiophysics University Hospitals of Strasbourg Strasbourg France

**Keywords:** biopsy, image‐guided, MRI, PET, prostate cancer, PSMA

## Abstract

Prostate cancer is one of the most common cancers among men, and accurate detection and diagnosis are crucial for effective treatment planning. Diagnostic prostate biopsy plays a pivotal role in the detection and characterization of prostate cancer. Recent advancements in molecular imaging, particularly with Positron Emission Tomography (PET) using radiolabelled Prostate‐Specific Membrane Antigen (PSMA) tracers, have shown significant improvements in enhancing prostate cancer detection. PSMA PET, when combined with magnetic resonance imaging (MRI) in hybrid PET/MRI systems, provides improved sensitivity and specificity, enabling more precise localization of clinically significant prostate cancer (csPCa) lesions. This narrative review explores the evolving role of PET/CT and PET/MRI‐guided prostate biopsy. We examine the integration of PET with MRI for the detection of prostate cancer, highlighting key studies that have demonstrated improved diagnostic outcomes. Additionally, we discuss the current limitations, including the high costs and longer scan times associated with PET/MRI, as well as the challenges in data interpretation. The review also considers emerging technologies, such as promising molecular probes for prostate PET imaging, such as gastrin‐releasing peptide receptor (GRPR) and fibroblast activation protein inhibitor (FAPI). Finally, the present work provides the clinician with a comprehensive yet concise up‐to‐date review of the literature to easily evaluate the possibilities currently offered by hybrid imaging technologies of personalized imaging‐guided biopsy for prostate cancer patients.

## Graphical abstract

Prostate biopsy plays a pivotal role in the detection and characterization of prostate cancer. PSMA PET‐targeted biopsy represents a significant advancement in prostate cancer diagnostics, offering higher sensitivity, specificity and accuracy in detecting clinically significant lesions compared to traditional methods. PSMA PET, when combined with mpMRI, eventually in hybrid PET/MRI systems, provides improved sensitivity and specificity, enabling more precise tumour localization and a more personalised approach to prostate cancer management.

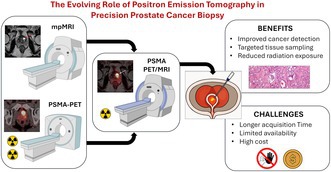

## 1 PROSTATE CANCER BIOPSY: AN OVERVIEW

Prostate carcinoma is one of the most common cancers in men worldwide, typically affecting older individuals. Clinically, prostate cancer often remains asymptomatic in its early stages, and many cases are detected incidentally through elevated serum prostate‐specific antigen (PSA) levels or imaging studies.^1^ Symptoms, when they occur, may include difficulty urinating, blood in the urine or pelvic discomfort. Diagnostic workup involves histopathological confirmation via prostate biopsy. Early detection through PSA testing and imaging has significantly improved the prognosis, allowing for surgery, radiotherapy and targeted therapies. Despite advances in treatment, prostate carcinoma remains a leading cause of cancer‐related morbidity and mortality, emphasizing the need for improved diagnostic and therapeutic tools.^2^

Diagnostic prostate biopsy plays a critical role in the detection and characterization of prostate cancer. It is essential for confirming diagnosis and determining the Gleason score, which helps guide treatment decisions and provides prognostic information. The most used biopsy method is the transrectal ultrasound (TRUS)‐guided biopsy.^3^ Accordingly, a 12‐core systematic biopsy, including apical and far‐lateral cores in the template distribution, is performed to optimize cancer detection and to reduce the need for repeat biopsies.^4^ However, TRUS lacks the ability to accurately visualize tumours, especially in certain areas of the prostate like the anterior or apical regions, which can lead to missed prostate cancer. Moreover, TRUS‐guided biopsy is not free of complications such as infection, including prostatitis or, in some cases, sepsis, despite prophylactic antibiotics. Other concerns are haematuria or rectal bleeding, discomfort or pain during the procedure and risk of urinary retention or damage to surrounding structures.

Recent advancements, including multiparametric MRI (mpMRI), have enhanced the accuracy of prostate biopsies in detecting clinically significant csPCa. mpMRI can help localize suspicious areas in the prostate, allowing for targeted biopsy, which reduces the risk of missing clinically significant prostate cancer (csPCa) and minimizes the likelihood of unnecessary biopsies in benign areas.^5^ The incorporation of mpMRI in the early diagnostic algorithm before the biopsy, as recommended in both the UK and the EU, has proven to be highly beneficial. A 2019 Cochrane review^6^ highlighted that the sensitivity for identifying csPCa was notably high (91%–95%), although specificity was considerably lower (35%–37%). MRI‐based diagnosis provides strong negative predictive value and sensitivity, reducing the need for unnecessary biopsies. These results have been similarly observed outside of formal clinical trials, despite variations in factors such as disease prevalence, MRI equipment and the expertise of involved medical professionals.^7^, ^8^, ^9^, ^10^, ^11^, ^12^ The PROMIS^13^ and PRECISION^14^ trials have demonstrated that conducting biopsies in all patients suspected of having prostate cancer without prior mpMRI leads to an increased rate of false‐positive diagnoses of csPCa. These studies have mainly contributed to clinical guidelines, including those from the European Association of Urology,^15^ which advocate for the use of mpMRI to help patient selection before prostate biopsy. Nevertheless, mpMRI has limitations in detecting csPCa, particularly in areas like the transition and central zones, which are often referred to as ‘blind spots’. mpMRI may miss 5%–10% of csPCa and can also underestimate tumour volume by up to three times.^16^, ^17^, ^18^ The necessity for accurate detection of aggressive prostate cancer has driven the development of additional diagnostic techniques, particularly to guide biopsy procedures more effectively. Molecular imaging, especially with radiotracers for positron emission tomography (PET) investigations, and the latest generations of PET/CT and PET/MR, has emerged as a critical tool in this regard.

Considering the above, the aim of this narrative review is to provide the clinician with a comprehensive yet concise up‐to‐date review of the literature to evaluate the diagnostic possibilities currently offered by molecular imaging technologies. Specifically, it focuses on PET tracers like radiolabelled Prostate‐Specific Membrane Antigen (PSMA) and their integration into biopsy‐guided diagnosis, supporting personalised management for prostate cancer patients while adapting the best choice to each patient's clinical situation.

## 2 PET‐GUIDED PROSTATE BIOPSY: WHERE WE STAND

By providing information on biological characteristics of prostate cancer cells, molecular imaging techniques enable clinicians to identify areas with increased metabolic activity or receptor expression, which often correlate with aggressive disease (Table 1). This precision allows for targeted biopsies, improving the detection of csPCa while reducing the likelihood of sampling errors associated with traditional biopsy methods. The development and incorporation of PET imaging into biopsy‐guided practices represent a significant advancement in prostate cancer management, ensuring more accurate diagnosis and better treatment planning.

**TABLE 1:** Comparison of clinically available radiotracers for prostate cancer imaging.

Radiotracer	Key Characteristics (physical half‐life)	Advantages	Drawbacks	Clinical Utility
[18F]Fluorocholine	Synthetic analogue of choline (110 min)	Widely accessible, useful for detecting metastases	Urinary excretion can obscure local lesions	Diagnostic alternative if PSMA is unavailable
[11C]Choline	Synthetic analogue of choline (20 min)	Minimal urinary excretion	Requires cyclotron availability, limited access	Limited to high‐resource centers
[68Ga]PSMA	Targets PSMA receptors (68 min)	High sensitivity for csPCa, detects metastases	Lower spatial resolution, Urinary excretion	Widely used in diagnosing and staging
[18F]PSMA‐1007	Targets PSMA receptors (110 min)	Better resolution, longer imaging window, minimal urinary excretion	Equivocal bone uptake may interfere with the detection of bone lesions	Effective for primary and recurrent cancer
[18F]DCFPyL	Targets PSMA receptors (110 min)	Better resolution, longer imaging window	Urinary excretion	Effective for primary and recurrent cancer

### 2.1 Choline PET

[^18^F]Fluorocholine and [^11^C]Choline have been widely used for PET imaging of prostate cancer, especially in patients with rising PSA levels after initial treatment, when conventional imaging modalities may fail to identify metastases.^19^ Both are synthetic analogues of choline, a precursor molecule for the synthesis of phosphatidylcholine, which is a key component of cell membranes. Prostate cancer cells exhibit increased choline metabolism due to their rapid proliferation, leading to elevated radiotracer uptake. Both tracers are useful in detecting primary prostate cancer, as well as nodal and distant metastases, offering improved detection over traditional methods, particularly when hybrid PET/MRI systems are used.^20^, ^21^

[^11^C]Choline is a radiotracer available only in medical centres with a cyclotron. Its key advantage is its short half‐life, which prevents urinary excretion, ensuring that urinary activity in the pelvic region does not interfere with imaging. This is particularly useful for accurate pelvic analysis. On the other hand, [^18^F]Fluorocholine is more widely accessible. While [^11^C]Choline and [^18^F]Fluorocholine yield similar results in detecting recurrence, meta‐analyses show that [^18^F]Fluorocholine is slightly more effective due to its longer half‐life, which allows for imaging 45–60 min after injection.^22^ This results in higher choline uptake in tumour cells over time, increasing tumour‐to‐normal tissue contrast. However, [^18^F]Fluorocholine has the drawback of urinary elimination, which can obscure local recurrence sites due to the presence of radiolabelled urine in the ureters and bladder.^23^ This can undermine the reliability of PET imaging in detecting small or focal lesions, ultimately compromising the accuracy of imaging‐guided prostate biopsy. To mitigate this issue, some protocols address this limitation by performing dynamic or early pelvic imaging, or administering diuretics before imaging to clear the bladder, enhancing the accuracy of the PET scan. However, these approaches are not universally adopted, and standardization across institutions remains a challenge.^24^ Over time, [^18^F]Fluorocholine has been used in clinical trials and has contributed to staging decisions, treatment planning and monitoring the response to therapy. However, with the emergence of more specific and sensitive tracers, the role of [^18^F]Fluorocholine has been somewhat diminished in prostate cancer imaging.^25^

A small study assessing the feasibility of [^11^C]Choline PET‐CT/TRUS fusion‐guided prostate biopsy in 15 men with persistently elevated PSA and negative mpMRI after a prior negative biopsy found that PET identified lesions in 14/15 patients (93.3%), with prostate cancer detected in 46.7% (7/15), including four cases of clinically significant disease (Gleason score >6). Despite high sensitivity, [^11^C]Choline PET exhibited low specificity. In a prospective trial with a larger patient cohort (56 patients), the effectiveness of [^18^F]Fluorocholine PET/mpMRI in improving risk stratification for MRI‐guided prostate biopsies was compared to mpMRI alone for detecting csPCa (Gleason ≥3 + 4). The study found that integrating [^18^F]Fluorocholine PET with mpMRI significantly improved diagnostic accuracy: lesion‐based accuracy increased from 67.8% (AUC .73) to 88.9% (AUC .90), while per‐patient accuracy improved from 70.0% (AUC .76) to 92.9% (AUC .93).^26^ This combined approach enhances risk stratification of intermediate‐risk lesions, potentially reducing unnecessary biopsies while improving the detection of aggressive cancers.^27^

Overall, [^18^F]Fluorocholine remains a valuable diagnostic tool, particularly in cases where radiolabeled PSMA‐based PET imaging is not available.

### 2.2 PSMA PET/CT

**FIGURE 1:**
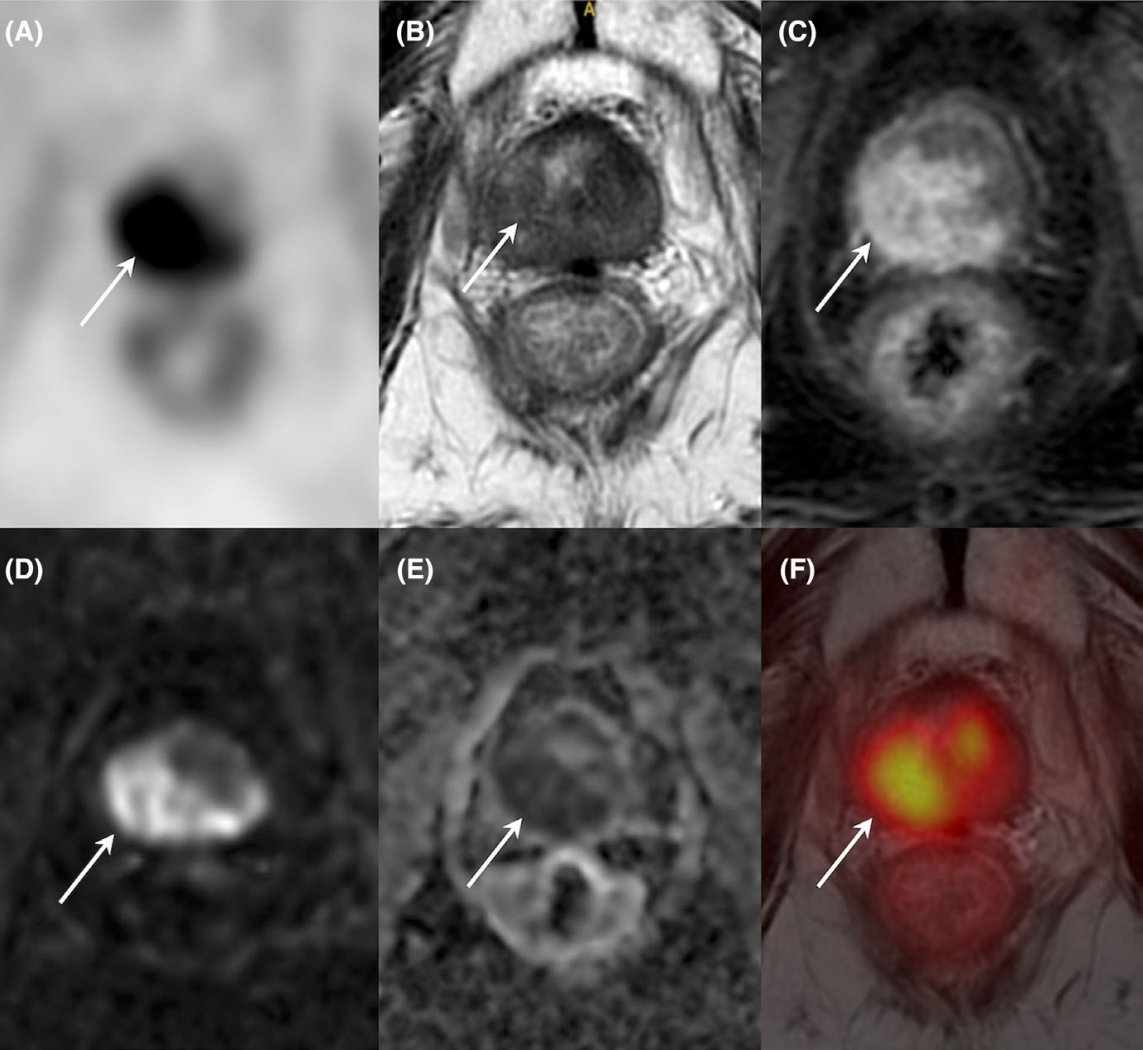
Typical results of [^68^Ga]PSMA PET/MRI imaging in a patient with prostate cancer (primary staging) developed in the peripheral zone of the right lobe and capsular infiltration (axial PET (A), T2‐weighted MRI (B), contrast‐enhanced MRI (C), Diffusion Weighted (DWI) MRI (D), Apparent Diffusion Coefficient (ADC) MRI (E), PET/MRI hybrid imaging (F)). Tumour appears as a 15 mm hypointense T2‐weighted lesion with high contrast enhancement, diffusion restriction, and intense [^68^Ga]PSMA uptake (arrows).

The breakthrough in prostate cancer imaging came with the advent of PET tracers targeting PSMA, which is highly expressed in prostate cancer. The expression level of PSMA is significantly higher in cancerous tissues compared to normal prostate tissues, making it an ideal target for both PSMA‐targeted molecular imaging and therapies.^28^, ^29^ PSMA PET‐guided biopsy uses PSMA as a molecular marker to enhance the precision of prostate cancer biopsy by utilizing prebiopsy PET images to guide the needle to areas of high PSMA expression. The introduction of PSMA PET for clinical use marked a significant milestone, providing superior sensitivity and specificity for detecting primary and recurrent prostate cancer compared to conventional imaging methods^30^, ^31^, ^32^ (Figure 1).

In studies evaluating PSMA PET, even in patients with previous negative biopsy results, PSMA PET has demonstrated exceptional performance with a 100% sensitivity and negative predictive value, 68.4% specificity and 80.6% accuracy in detecting csPCa. These findings underscore PSMA PET's ability to detect csPCa lesions even when other methods have failed.^33^ The first prospective study assessing the feasibility of [^68^Ga]PSMA617 PET/CT‐guided biopsy evaluated men with previous negative biopsies but persistent clinical suspicion of prostate cancer. The study revealed that PET/CT‐guided biopsy detected csPCa in 39% of patients, compared to 32% for TRUS‐guided biopsies. When both methods were combined, detection increased to 67% in patients with positive [^68^Ga]PSMA617 PET results, whereas neither targeted nor systematic biopsy detected clinically significant cancer in patients with negative [^68^Ga]PSMA617 PET results. Accordingly, in challenging cases with persistently elevated PSA levels despite negative prior biopsy results, PSMA PET‐guided biopsy has proven to be invaluable.^34^ Furthermore, PSMA PET/CT‐guided biopsies have shown a significantly higher detection rate for csPCa with a Gleason score of 7 (3 + 4) or higher compared to traditional systematic biopsies.^35^ Specifically, PSMA PET‐guided biopsy performed with an SUVmax cutoff value of 8 demonstrated higher sensitivity (100% vs. 90.9%), specificity (80.3% vs. 78.9%) and accuracy (92% vs. 86.2%) than mpMRI‐targeted biopsies.^36^ In a recent multicenter observational cohort study including 72 patients, PSMA PET/CT‐guided biopsy was more effective in biopsy‐naïve patients compared to those undergoing repeated biopsies.^37^ The uncertainty regarding the need for standard biopsy in combination with PET‐targeted biopsy has been recently investigated. While PSMA PET‐guided biopsy can simplify the interventional procedure and reduce its invasiveness by decreasing the number of cores, standard biopsy seems not to be entirely replaced due to potential errors in the PET‐targeted procedure negatively influenced by limited spatial resolution and fusion inaccuracies.^38^ The ongoing DEPROMP trial^39^ will firstly evaluate the impact of adding PSMA PET/CT‐guided prostate biopsy to standard of care methods (systematic biopsy and MR‐guided biopsy) in 230 biopsy‐naïve patients providing data on tumour detection, risk stratification and the need for multiple biopsies. Results will help assess the value of PET/CT‐guided biopsy in treatment decisions and tumour staging.

[^68^Ga]PSMA11 PET/CT was used to guide prostate biopsies through the gluteal muscle, identifying clinically significant prostate cancer in 80% of cases, compared to 25% with standard TRUS‐guided biopsy. Notably, by omitting biopsies in PET‐negative patients, only 6% of csPCa would have been missed. The absence of adverse events with the trans gluteal biopsy technique further underscores its safety, in contrast to haematuria, urine retention and infection observed in the TRUS‐guided group.^40^

More recently, [^18^F]PSMA ligands have been proposed, such as [^18^F]DCFPyL and [^18^F]PSMA‐1007, offering several advantages over [^68^Ga]PSMA due to the lower positron range of [^18^F] compared to [^68^Ga], which results in higher spatial resolution, and their longer half‐life (110 min compared to 68 min for [^68^Ga]). This longer half‐life allows for improved tumour‐to‐background ratios during delayed imaging, providing a better imaging window. Moreover, nonurinary excretion of [^18^F]PSMA‐1007 might present some advantage regarding delineation of local disease. In a prospective proof‐of‐concept study, the intraoperative quantification of [^18^F]PSMA‐1007 PET/CT uptake in core biopsies was assessed for accurate lesion sampling in prostate cancer.^41^ Five patients with suspected prostate cancer underwent [^18^F]PSMA‐1007 PET/CT scans, followed by PET/CT‐guided biopsy. The activity in biopsy cores was measured and correlated with histopathology, tumour length and PSMA expression. Results showed significantly higher counts in needles with prostate cancer compared to those without, with a sensitivity of about 65% and specificity of 87% for detecting prostate cancer at a threshold of 75 cpm. These findings confirm accurate lesion sampling intraoperatively, potentially reducing the need for saturation biopsy. The DeTeCT trial evaluated the performance of [^18^F]DCFPyL PET/CT for identifying primary prostate cancer, forecasting a 93% detection rate for csPCa, with an 87% detection rate.^42^ These innovations have led to PSMA PET‐based imaging becoming increasingly integrated into clinical practice and guidelines, representing a major leap forward in precision oncology for prostate cancer.^43^

### 2.3 PSMA PET/MRI

**FIGURE 2:**
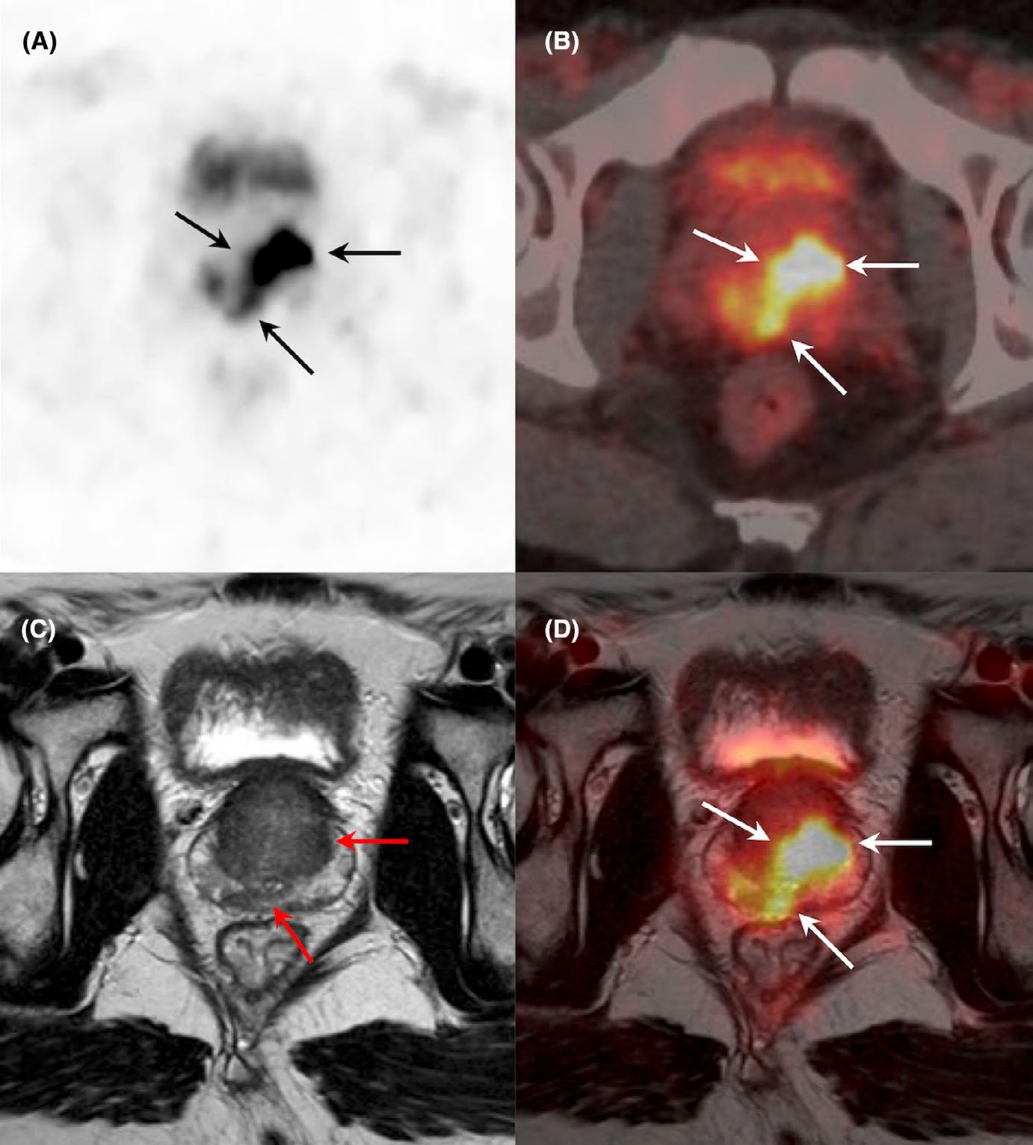
Initial staging in patient with biological suspicion of prostate cancer (PSA: 53.4 ng/mL). [^18^F]PSMA1007 PET/CT and MRI imaging (axial PET (A), PET/CT (B), T2‐weighted MRI (C), PET/MRI fused images (D)) demonstrated intense pathological uptake in the bilateral peripheral zone, mostly involving the left anterior, posterolateral and posteromedial zones, with invasion of the seminal vesicle (MRI PI‐RADS: 5, SUVmax: 20.8). [^18^F]PSMA1007 PET also showed tumoral extension in the transitional zone, which was not revealed by MRI. Pathological examination after biopsy confirmed the presence of acinar adenocarcinoma with lymphovascular invasion and Gleason score: 7 (3 + 4).

PET/MRI combines the functional imaging capabilities of PET with the high‐resolution anatomical imaging of MRI, offering a comprehensive tool for prostate cancer diagnosis (Figure 2). The combination of PET and MRI data can provide more accurate localization of tumours in a single session. Though PSMA PET/CT‐guided biopsies are also considered reliable, the preferred imaging modality for PSMA PET‐guided biopsy remains PET/MRI.^44^ One of the benefits of PET/MRI over traditional PET/CT is its ability to reduce patient exposure to radiation by approximately 80%.^45^, ^46^ This reduction in radiation is a significant advantage, especially as more patients with prostate cancer undergo regular imaging for monitoring treatment response or recurrence (Table 2).

**TABLE 2:** Main advantages and drawbacks of PET/MRI for prostate cancer management.

Advantages	Drawbacks
Superior Soft Tissue Contrast: MRI provides excellent soft tissue resolution, which is enhanced when combined with PET for prostate cancer diagnosis.	Higher Costs: PET/MRI is more expensive compared to PET/CT, which may limit its use in routine clinical settings.
Multiparametric Imaging: PET/MRI integrates anatomical (MRI) and functional (PET) data, offering a comprehensive assessment of tumour biology.	Longer Acquisition Time: PET/MRI scans take longer, which may impact patient compliance and reduce throughput in clinical settings.
Simultaneous Data Acquisition: PET and MRI data can be collected in a single session, offering a ‘one‐stop‐shop’ diagnostic process.	Complex Protocols: The imaging protocols for PET/MRI are more complex, requiring skilled personnel for accurate execution.
Reduced Radiation Exposure: PET/MRI offers significantly lower radiation exposure compared to PET/CT (up to 80% reduction).	Data Interpretation Challenges: The larger volume of data generated by PET/MRI can make the interpretation more time‐consuming and complex.
Enhanced Tumour Detection and Staging: PET/MRI is particularly useful in detecting small lesions and providing accurate staging of prostate cancer, including metastatic spread.	Slow Adoption and Integration: PET/MRI adoption has been slow due to high costs, complex workflows and the lack of established protocols in clinical practice.
Improved Patient Management: PET/MRI has shown to influence treatment decisions and improve management of prostate cancer, including changes in therapy based on findings.	Limited Whole‐Body Staging: PET/MRI is less efficient for whole‐body staging compared to PET/CT due to its slower acquisition time.

**FIGURE 3:**
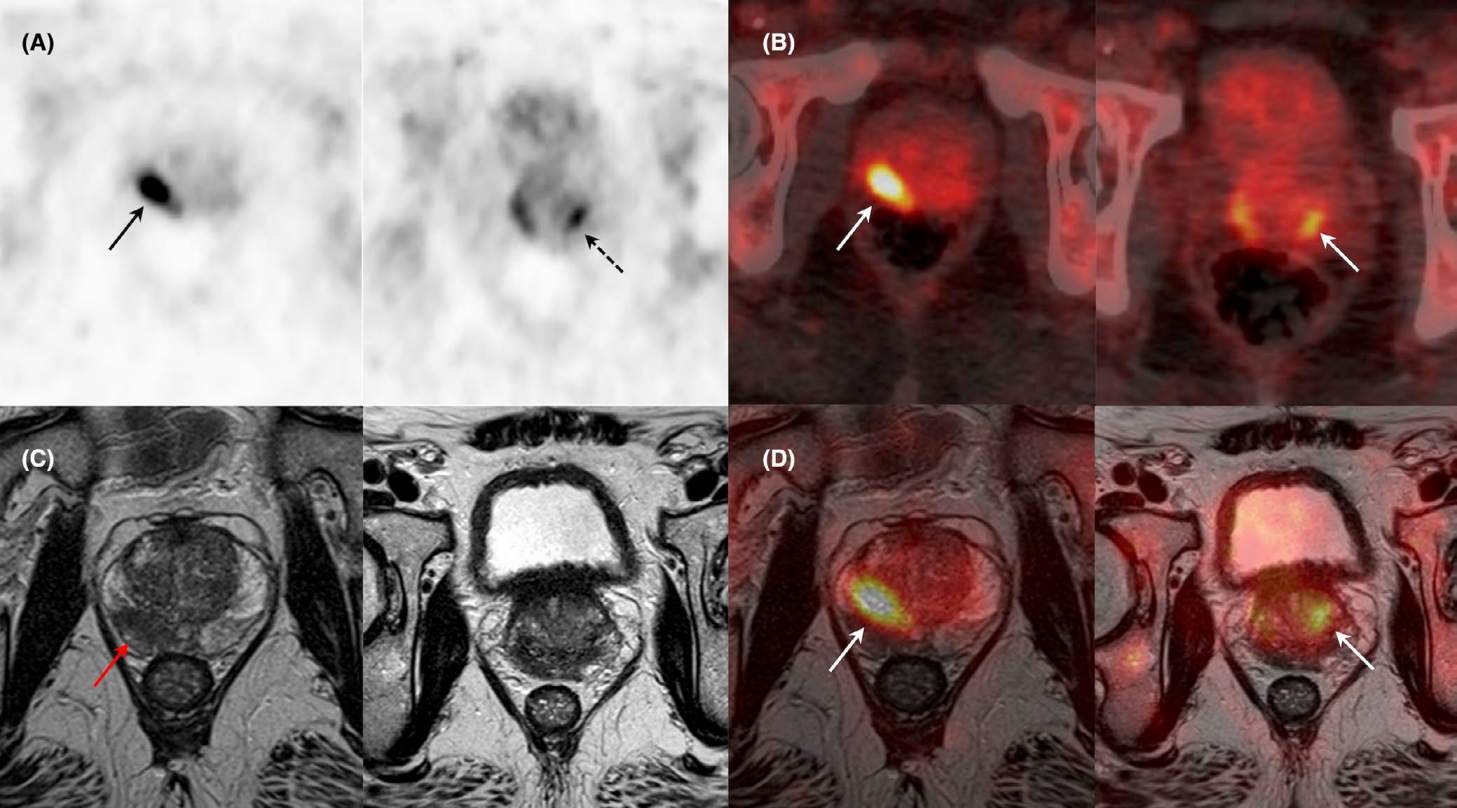
Results of [^18^F]PSMA1007 PET/CT and MRI imaging (2‐levels axial PET (A), PET/CT (B), T2‐weighted MRI (C), PET/MRI fused images (D)) in a patient with biological suspicion of prostate cancer (PSA: 9 ng/mL). Both PET and MRI showed a pathological lesion, involving the posterolateral and posteromedial right peripheral zone (19 × 13 × 8 mm, SUVmax 13, MRI PI‐RADS: 5). An additional [^18^F]PSMA1007 focal uptake (SUVmax 8.56) was revealed in the left transitional zone. Pathological examination after biopsy confirmed the presence of acinar adenocarcinoma (both lesions), Gleason score 7 (4 + 3).

PSMA PET/MRI has been shown to enhance the diagnostic accuracy for prostate cancer, particularly in detecting lesions missed by conventional imaging techniques. The integration of PSMA PET with MRI has been shown to increase sensitivity and negative predictive value, allowing clinicians to better discriminate between clinically significant and indolent disease.^47^ The combination of PSMA PET and MRI improved the detection rate of clinically significant disease (Figure 3), providing substantial guidance in treatment decisions, particularly in patients with biochemical recurrence after primary therapy.^48^ The use of PSMA PET/MRI in detecting early‐stage prostate cancer and recurrent disease has been proven to be effective, as it offers the possibility to accurately assess small lesions and metastatic sites, often undetectable by traditional methods. Furthermore, PSMA PET/MRI offers the potential for reducing unnecessary biopsies, suggesting that these patients may be better managed through active surveillance.^40^ This could significantly reduce the number of invasive procedures and provide a more tailored treatment approach.

**FIGURE 4:**
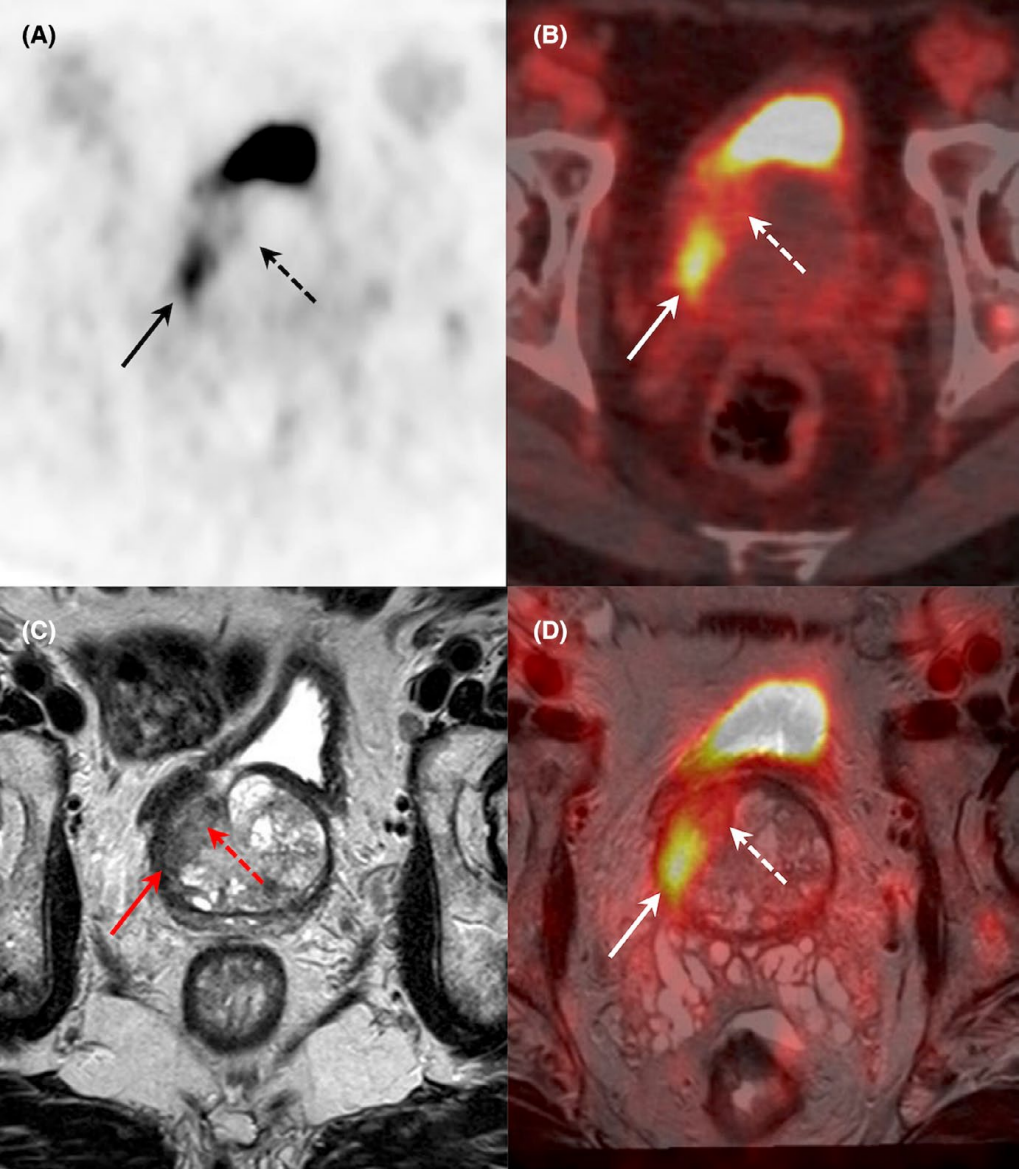
Results of [^18^F]DCFPyL PET/CT and MRI imaging (axial PET (A), PET/CT (B), T2‐weighted MRI (C), PET/MRI fused images (D)) in a patient with prostate cancer biological suspicion (PSA: 18.6 ng/mL). MRI revealed a tumoral lesion of 25 × 21 × 18 mm in the posterior part of the right transitional zone (MRI PI‐RADS: 5). Intense [^18^F]DCFPyL focal uptake (SUVmax: 12) was shown exclusively in the posterior part of the lesion. Pathological examination after biopsy confirmed the presence of acinar adenocarcinoma, Gleason score: 7 (4 + 3). After surgery, tumour was localized only in the PET‐positive lesion, with extracapsular invasion.

A recent meta‐analysis evaluated the diagnostic accuracy of PSMA PET‐targeted biopsy for detecting prostate cancer and the impact of the combination of PSMA PET and MRI^49^ including five prospective studies with a total of 497 patients who underwent PSMA PET. PSMA PET could potentially reduce unnecessary biopsies in patients with negative PSMA PET results, providing a more accurate risk stratification for patients with suspected prostate cancer. Pooled sensitivity was 89%, specificity 56%, positive predictive value of 69% and negative predictive value of 78%. The combination of PSMA PET and MRI further improved the sensitivity (91%) and negative predictive value (85%) compared to PSMA PET alone. The PRIMARY trial, a prospective multicenter study, evaluated the added value of pelvic PSMA PET/CT in conjunction with standard mpMRI for prostate cancer biopsy. This combined approach improved sensitivity (97% vs. 83%) and negative predictive value (91% vs. 72%) for csPCa compared to mpMRI alone^48^ (Figure 4).

A prospective single‐center study had compared [^68^Ga]PSMA11 PET/MRI‐guided biopsy with standard template biopsy in biopsy‐naïve patients. The study found that PET/MRI achieved 90% accuracy for detecting csPCa, with high sensitivity (96%) and specificity (81%), while PET‐guided biopsy showed a reduced accuracy of 71%.^44^

Another recent prospective study, including 55 patients with solitary PET‐positive prostate lesions, assessed the feasibility and diagnostic performance of [^18^F]DCFPyL PET/CT‐ultrasound (PET/CT‐US) or PET/MRI‐ultrasound (PET/MRI‐US) fusion targeted biopsies.^50^ A total of 178 biopsy cores were performed, with 82% of the cores being positive for prostate cancer, and 85.5% of those cases were clinically significant. Of the 55 patients, about 93% were diagnosed with prostate cancer, and 85.5% had csPCa. For patients who underwent PET/CT and PET/MRI, fusion‐targeted biopsies showed high detection rates, respectively 87.5% and about 83%. Among the nine patients who underwent both [^18^F]DCFPyL PET/CT and PET/MRI, seven showed abnormal MR signals in the PET‐positive lesions, with pathology confirming prostate cancer in all cases. The remaining two patients exhibited normal MR signals in the PET‐positive lesion areas, and subsequent biopsy revealed benign prostatic hyperplasia and prostatitis.

The PRIMARY Score for prostate cancer is designed to evaluate the aggressiveness of the disease by analysing various pathological features. It integrates crucial elements such as the Gleason score, PSA levels and MRI findings, providing a comprehensive assessment of the tumour's biological behaviour.^51^ Recent research shows that using intraprostatic patterns and intensity from [^68^Ga]PSMA PET/CT could be very promising, providing accurate results for detecting clinically significant prostate cancer (csPCa). However, more studies are needed before it can be widely used in clinical practice.^52^

**FIGURE 5:**
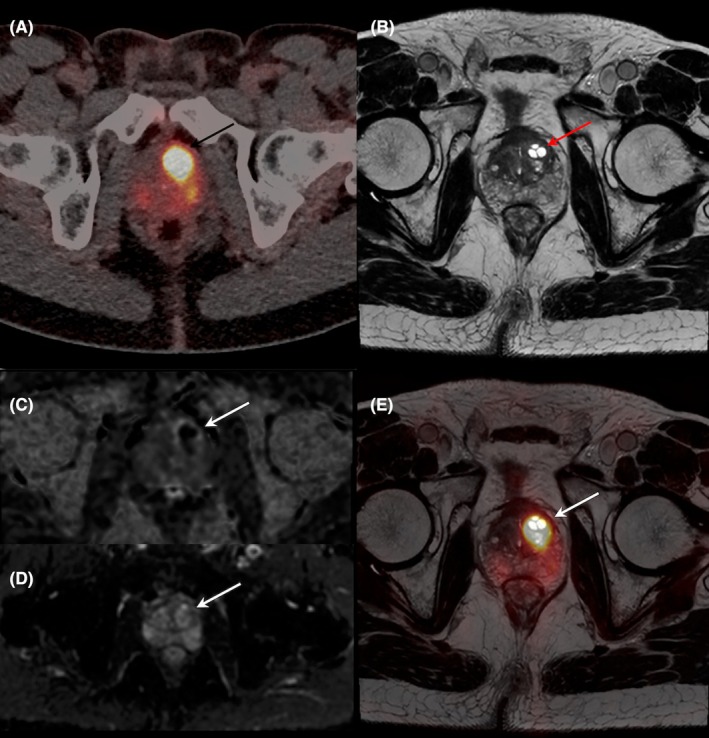
False positive results of [^18^F]DCFPyL PET/CT in a patient presenting with dysuria, without evidence of urinary tract infection, and repeated PSA levels above 20 ng/mL (axial PET/CT (A), T2‐weighted MRI (B), diffusion B sequence (C), ADC sequence (D), PET/MRI fused images (E)). PET/CT revealed two foci of increased uptake of [^18^F]DCFPyL, without any suspicious lesions in the prostate gland on mpMRI. Systematic and targeted transrectal prostate biopsies were performed, and all tissue samples were negative for prostate cancer. Final diagnosis after pathological examination was prostatic adenoma.

While PSMA PET/MRI offers significant advantages in detecting csPCa, it also has several limitations that should be considered (Table 2). One of the major drawbacks is its high cost, which remains an obstacle for a wide clinical utilization of PET/MRI, reducing its accessibility in resource‐limited settings. Moreover, one significant challenge in adopting PET‐guided biopsy across various institutions is the lack of standardized imaging protocols. Variations in equipment, radiotracers and imaging techniques can lead to inconsistent results, impacting the diagnostic accuracy of PET‐guided procedures. The longer scan times associated with PET/MRI can be challenging for patient compliance, especially in busy clinical environments.^47^ The complexity of scan protocols for PET/MRI, which involve both MRI and PET imaging sequences, necessitates a higher level of expertise from imaging specialists to ensure the accurate execution and interpretation of the procedure. Additionally, PSMA PET can produce false positives in benign conditions such as prostatitis or benign prostatic hyperplasia (BPH), which may lead to unnecessary biopsies or treatment interventions (Figure 5). Furthermore, the lack of specificity in certain clinical contexts, coupled with the need for advanced imaging infrastructure, means that PSMA PET/MR may not be a possible choice in all patient populations.

## 3 PET‐GUIDED PROSTATE BIOPSY: CLINICAL OVERALL CONSIDERATIONS

PET‐guided biopsy, particularly using PSMA, offers a transformative approach to improving prostate cancer diagnostics and treatment strategies. By integrating PET imaging, which highlights areas of high functional activity, with traditional biopsy methods, this technique enables precise localization of csPCa. This is especially valuable in challenging cases where conventional imaging may fall short. For instance, PSMA PET‐guided biopsy has demonstrated high sensitivity and accuracy, detecting csPCa even in patients with prior negative biopsies or persistently elevated PSA levels.

The utility of PET‐guided biopsies extends beyond just detection. By targeting the areas of the prostate with the highest expression of PSMA, the biopsy procedure becomes more efficient, reducing the need for unnecessary biopsies and limiting the risks associated with invasive procedures. This precision not only enhances the likelihood of identifying aggressive cancers but also helps in the management of patients with biochemical recurrence, guiding tailored treatments more effectively. PET/MRI fusion‐guided biopsy has demonstrated better sensitivity and specificity for detecting csPCa compared to traditional methods.

In addition to staging, PET imaging, when used in conjunction with mpMRI, provides complementary functional and anatomical information, leading to a more comprehensive assessment of primary tumour extent and potential local metastases. The integration of PET/MRI into treatment strategies could enhance the ability to guide focal therapies, such as High‐Intensity Focused Ultrasound (HIFU) or cryotherapy, offering targeted treatment options with fewer side effects compared to traditional whole‐gland treatments. The combination of PET's functional imaging with MRI's anatomical resolution allows for real‐time evaluation of treatment efficacy, ensuring that adjustments can be made early in the therapeutic process.

However, despite its clear advantages, the widespread adoption of PET‐guided biopsy is hindered by several challenges, notably the high cost and longer scan times associated with PET/MRI. These factors can limit its availability, particularly in resource‐limited settings. Additionally, the complexity of data interpretation and the need for highly trained personnel to manage the imaging protocols further complicate its integration into routine clinical practice. Despite these challenges, PET‐guided biopsy, especially when used alongside other imaging techniques like mpMRI, represents a step forward in precision oncology, improving outcomes and minimizing unnecessary procedures.

As further advancements in PET technology and radiopharmaceutical development continue, the role of PET‐guided treatment in prostate cancer management is expected to expand, offering more personalized and effective care for patients.

## 4 WHAT'S NEXT IN PET‐GUIDED PROSTATE BIOPSY

A recent study evaluated the targeting accuracy for prostate cancer detection of a novel PSMA radiotracer, [^64^Cu]DOTA‐PSMA‐3Q, also exploring the potential for real‐time lesion verification during surgery using radiation‐guided technology.^53^ Ten patients suspected of having prostate cancer underwent PET/CT imaging and received targeted biopsy combined with standard template biopsy 24 h after radiotracer injection. Intraoperative radioactivity of biopsy tissues was measured using a gamma spectrometer. PET/CT imaging 2 h post‐injection visualized all PSMA‐positive lesions. Moreover, of 132 biopsy cores, 53 were confirmed as prostate cancer, with significantly higher radioactivity in cancerous tissues compared to normal tissues (ROC AUC of .88). These findings suggest the potentiality of radiation‐guided technology to enhance the reliability of PSMA PET‐guided biopsy, providing immediate confirmation of lesion presence during surgery, and could improve biopsy outcomes in prostate cancer diagnosis.

Although PSMA is a useful target, its expression is not entirely specific to prostate cancer, which may result in false positives. Moreover, its absence in certain tumour lesions can lead to false negatives.^54^, ^55^, ^56^, ^57^ Recently, alternative biological targets have been identified, such as gastrin‐releasing peptide receptor (GRPR), which is overexpressed in several cancers, including prostate cancer, particularly in earlier stages. The integration of GRPR‐ and PSMA‐based dual‐tracer PET/CT may enhance diagnostic accuracy, reducing unnecessary biopsies by about 53% while achieving a detection rate of 77% without csPCa misdiagnosing.^58^ Additionally, [^68^Ga]GRPR PET/CT proved particularly effective in detecting and localizing primary prostate cancer, complementing mpMRI findings. Importantly, GRPR expression appears independent of PSMA expression, reinforcing the complementary value of GRPR‐ and PSMA‐targeted PET imaging.^59^ An in vivo trial further demonstrated that combining tracers targeting both GRPR and PSMA provides superior delineation of total tumour volume in prostate cancer patients.^60^ While GRPR‐based imaging is particularly beneficial for detecting low‐grade prostate cancer and lesions unaffected by hormonal treatment, PSMA imaging is more effective in high‐grade prostate cancer and post‐hormone therapy cases. Thus, GRPR imaging could serve as a valuable adjunct, especially for PSMA‐negative tumours.^61^, ^62^

Androgen receptor (AR) PET imaging has recently emerged as a valuable tool in prostate cancer research and management. Preliminary studies have shown an inverse relationship between PSMA expression and AR signalling, with AR inhibition leading to increased PSMA uptake in both preclinical models and patients.^63^, ^64^, ^65^

Other emerging imaging approaches involve somatostatin receptor (SSTR)‐targeted PET/CT. SSTR expression varies across prostate cancer subtypes. Savelli et al.^66^ reported heterogeneous SSTR expression in castration‐resistant prostate cancer (CRPC) metastases using [^68^Ga]DOTANOC PET/CT, while Alonso et al. suggested ^68^Ga‐DOTATATE PET/CT as a promising tool for visualizing SSTR expression in advanced metastatic prostate cancer to guide targeted therapies,^67^ particularly in cases of neuroendocrine differentiation.

Recent research has also investigated fibroblast activation protein inhibitor (FAPI) PET imaging as a promising alternative for prostate cancer detection, particularly in cases with low PSMA expression. Ortolan et al.^68^ found that [^68^Ga]FAPI PET effectively detected primary prostate cancer, while a systematic review by Hagens et al.^69^ demonstrated its ability to visualize both local and metastatic disease in genitourinary malignancies, including PSMA‐negative prostate cancer. Ergul et al.^70^ further reported that [^68^Ga]FAPI PET identified disease in 54% of PSMA‐negative prostate cancer cases, suggesting its potential as a complementary imaging modality. At present, [^68^Ga]FAPI PET has been mainly tested in metastatic disease settings, with limited information available for prostate‐guided biopsy. Overall, the development of PSMA‐alternative PET tracers reflects the need for more comprehensive and tailored approaches to prostate cancer detection. These emerging modalities offer complementary advantages to PSMA PET, addressing its limitations and improving the accuracy of diagnosis and disease characterization.

Advancements in artificial intelligence (AI) and radiomics are poised to significantly enhance the capabilities of PET in prostate cancer diagnostics.^71^ Radiomics involves extracting quantitative data from medical images, such as texture, shape and intensity features, which can be used to characterize tumours more accurately than conventional imaging alone. These features can provide insights into tumour heterogeneity, aggressiveness and potential treatment response, offering a more comprehensive assessment than traditional methods. AI, particularly machine learning algorithms, can analyse large datasets of radiomic features, helping to identify patterns that might be missed by human clinicians. These AI models can predict tumour behaviour, classify cancerous lesions and even guide treatment decisions by integrating both functional (PET) and anatomical (MRI) data. In prostate cancer, AI‐driven approaches can aid in detecting csPCa, improve risk stratification and reduce unnecessary biopsies by accurately distinguishing between indolent and aggressive tumours. The integration of AI and radiomics with PET/MRI could lead to a more personalized, data‐driven approach to prostate cancer diagnosis, further refining lesion detection and staging. As these technologies evolve, their clinical implementation is expected to improve diagnostic accuracy, minimize errors in image interpretation and help clinicians tailor treatment plans to individual patients. However, although technological advances in automation and AI have made strides in addressing some of these challenges, the full clinical integration of AI into PET/MRI workflows remains at early stages.^72^, ^73^

## 5 CONCLUSION

PSMA‐targeted biopsy represents a significant advancement in prostate cancer diagnostics, offering higher sensitivity, specificity and accuracy in detecting clinically significant lesions compared to traditional methods. The integration of PSMA PET with mpMRI and advanced biopsy techniques enhances diagnostic outcomes and allows for a more personalized approach to prostate cancer management. While challenges such as cost, accessibility and the potential for false positive or negative results remain, PSMA PET continues to show great promise in improving diagnosis, particularly in cases where conventional imaging and biopsy results are inconclusive. As technological advancements, including artificial intelligence and radiomics, evolve, PSMA PET/MRI is expected to further refine prostate cancer diagnosis and treatment, leading to more tailored and effective management strategies for patients.

## AUTHOR CONTRIBUTIONS

Alessio Imperiale and Valentina Berti contributed to the review conception and design. All authors substantially contributed to material preparation, data collection, and analysis. The first draft of the manuscript was written by Alessio Imperiale, and all authors commented on previous versions of the manuscript. All authors read and approved the final manuscript.

## FUNDING INFORMATION

The authors declare that no funds, grants or other support was received during the preparation of this manuscript.

## CONFLICT OF INTEREST STATEMENT

The authors have no relevant financial or nonfinancial interests to disclose.

## CONSENT TO PARTICIPATE

This is not necessary for reviews.

## Data Availability

This is not necessary for reviews.
